# Nanomachining-enabled strain manipulation of magnetic anisotropy in the free-standing GaMnAs nanostructures

**DOI:** 10.1038/s41598-019-50115-1

**Published:** 2019-09-20

**Authors:** Chanuk Yang, Jae-Hyun Lee, Myunglae Jo, Hyung Kook Choi, Seondo Park, Young Duck Kim, Sung Un Cho, Donguk Kim, Yun Daniel Park

**Affiliations:** 1 Department of Physics & Astronomy and Institute of Applied Physics, Seoul National University, Seoul, 08826 Korea; 2 Service de Physique de l’Etat Condensé, CEA, CNRS, Université Paris Saclay, CEA Saclay, 91191 Gif sur Yvette cedex, France; 3 Department of Physics, Research Institute of Physics and Chemistry, Chonbuk National University, Jeonju, 54896 Korea; 4 Department of Physics, Kyung Hee University, Seoul, 02447 Korea; 5 Center for Quantum Coherence in Condensed Matter, Korea Advanced Institute of Science and Technology, Daejeon, 305-701 Korea; 6 Present Address: Component Solution Business Unit, Samsung Electro-Mechanics, Suwon, 16674 Korea

**Keywords:** Ferromagnetism, Spintronics

## Abstract

Strain perturbs atomic ordering in solids, with far-reaching consequences from an increased carrier mobility to localization in Si, stabilization of electric dipoles and nanomechanical transistor action in oxides, to the manipulation of spins without applying magnetic fields in n-GaAs. In GaMnAs, a carrier-mediated ferromagnetic semiconductor, relativistic spin-orbit interactions – highly strain-dependent magnetic interactions – play a crucial role in determining the magnetic anisotropy (MA) and anisotropic magnetoresistance (AMR). Strain modifies the MA and AMR in a nanomachined GaMnAs structure as measured by the anomalous Hall effect (AHE) and the planar Hall effect (PHE). Here, we report an MA modification by strain relaxation in an isolated GaMnAs Hall bar structure and by applying a range of local strains via fabricating asymmetrically mechanically buckled GaMnAs micro-Hall bar structures. In the AHE and PHE measurements, we observe a reduction in the in-plane MA and an enhancement in the out-of-plane MA as the compressive strain due to the lattice mismatch relaxes in the suspended structure. The functionality of such mechanical manipulation, as well as the two-level mechanical state and the corresponding AHE responses, is demonstrated by a fully scalable binary mechanical memory element in a GaMnAs single Hall cross structure.

## Introduction

Carrier-mediated ferromagnetic semiconductors are promising candidate materials for spintronic devices^1^. The beneficial characteristics unique to semiconductors and ferromagnets are advantageously entwined, in particular adding innovative functionalities to existing device applications. The interplay between electronic charge and spin has been meticulously demonstrated by a direct correspondence between carrier concentration and ferromagnetic ordering – either statically controlled by growth parameters and post-growth annealing^1^ or dynamically controlled by utilizing electrical fields^2^. Mechanical strain can also induce changes in the electronic band structure and spin-orbit interactions (SOIs)^3–7^. As a result, in III-V-based ferromagnetic semiconductors, properties such as magnetic anisotropy (MA), the intrinsic anomalous Hall effect (AHE) and the planar Hall effect (PHE) are highly sensitive to changes in strain^8–15^.

Relativistic effects that lead to SOIs are potentially more applicable for spintronics; however, they are generally inconsequential in determining the onset of magnetic ordering^1^. Indeed, such a strain dependence has been employed to demonstrate its applicability for low-power logic^16^ and memory devices^17,18^, both by taking advantage of changes in the MA, with marginal effects on the ferromagnetic ordering temperatures^19^. The intrinsic anomalous Hall Effect^20–23^ is also strongly dependent on strain, with an expected halving of the anomalous Hall conductivity (*σ*_*xy*_) with an increase in the compressive strain of less than one percent^22^. With the typical *σ*_*xy*_ of GaMnAs being on the order of 10 Ω^−1^cm^−1^, changes due to minute variations in the strain from micron-sized Hall bar structures would possess large signal-to-noise ratios befitting low-dissipation device applications. Furthermore, the planar Hall effect^9^ measurement is a powerful tool for investigating the in-plane magnetic anisotropy related to the strain of the lattice.

Nanomechanical structures of GaMnAs^13–15,24^ allow an outstanding opportunity to manipulate and observe the dynamics of such an interplay. Here, we demonstrate the strain dependencies of the PHE and AHE in GaMnAs by utilizing mechanically isolated micro-sized Hall bar structures. Furthermore, we investigate the strain modulation of the AHE in nanomachined GaMnAs buckled Hall bar structures.

## Experiment and Discussion

### Freely suspended strain-relaxation structure

**Figure 1:**
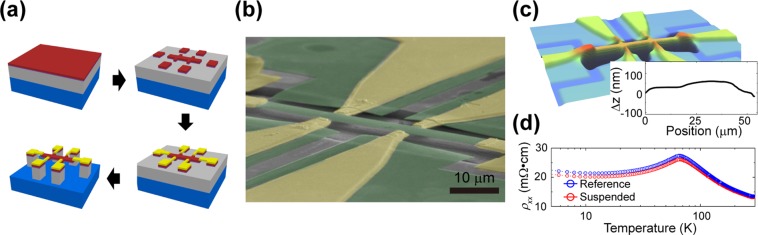
Freely suspended strain-relaxation structure. The freely suspended GaMnAs Hall bar structure designed for strain relaxation is realized by micromachining techniques. (**a**) Schematic of the fabrication processes. (**b**) SEM image of a freely suspended structure with added false colour to distinguish the GaMnAs layer (green) and electrical leads (gold). The absence of a GaMnAs layer beneath the electrical leads isolates the freely suspended GaMnAs Hall bar structure from extrinsic strain. (**c**) Profile image obtained by a non-contact optical profilometer and a height profile of the suspended GaMnAs Hall bar structure (inset). The deformation is confirmed to be less than 100 nm over a length of 60 μm during the fabrication. (**d**) The temperature dependences of the longitudinal resistivity (*ρ*_*xx*_ vs. *T*) indicate that all the structures have similar magnetic ordering temperatures (*T*_*C*_), whether they rest on the AlGaAs layer (blue) or are freely suspended (red).

The freely suspended GaMnAs Hall bar structure designed for strain relaxation is grown by low-temperature molecular beam epitaxy (LT-MBE): GaMnAs (100 nm)/GaAs (80 nm)/AlGaAs (2000 nm) is grown on a SI-GaAs (001) substrate with ~6% Mn concentration in the GaMnAs epilayer and ~75% Al concentration in the AlGaAs sacrificial layer. The details of the LT-MBE growth of the GaMnAs epilayer and characterization of the epilayer have been described elsewhere^25,26^. A 60-µm-long and 6-µm-wide freely suspended GaMnAs Hall bar structure with a reference structure (identical shape and dimension, but not suspended) is patterned in a single specimen by e-beam lithography, as shown in Fig. 1(a). Then, the Hall bar structures are defined by citric acid/hydrogen peroxide etching with a volume ratio of 7:2 (etch rate: ~200 nm/min), isolating the Hall bar from the remaining GaMnAs layer. Then, metal electrodes (Au 100 nm/Cr 80 nm) are deposited by standard e-beam lithography and e-beam deposition. The thickness of the metallic layer is chosen so that the deposited metal covers the step edge at the end of the GaMnAs structure from the GaMnAs etch. Selective etching of the AlGaAs layer using 10% HF is utilized to suspend the Hall bar structure, while the reference structure is protected from etching by e-beam resist. After the definition of the structure, the critical point drying method is applied to prevent a possible structural collapse. Figure 1(b) shows an SEM image of the realized freely suspended Hall bar structure, which is completely suspended from the substrate. The GaMnAs layer is isolated from the substrate and clamped by soft metal electrodes, without any GaMnAs beneath, solely supporting the Hall bar structure.

The quality of the freely suspended Hall bar structure is demonstrated by both optical and electrical measurements. As shown in Fig. 1(c), the suspended Hall bar is examined by a non-contact optical profilometer (NANO View-E1000) for any possible physical deformation during the fabrication process. The realized suspended Hall bar structure demonstrates little deformation of less than 100 nm along 60 µm. For further verification, the temperature dependence of the resistivity is plotted for both the free-standing Hall bar structure and the reference structure (Fig. 1(d)). All magnetotransport measurements are performed in a closed-cycle magnetocryostat (IceOxford DRYICE4 TL) using the standard AC lock-in technique (*I* = 0.1 µA at 17 Hz and 27 Hz). Both structures clearly exhibit typical behaviours of ferromagnetic semiconductors and identical temperature-dependence anomalies (resistance peak) corresponding to *T*_*C*_ ~ 65 K, indicating that the fabrication process causes no significant degradation and does not alter the macroscopic electrical characteristics of the GaMnAs epilayer.

### Magnetotransport measurements in the freely suspended strain-relaxation structure

**Figure 2:**
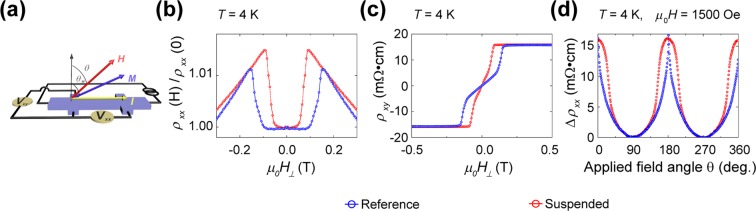
Anomalous Hall effect measurement in the freely suspended strain-relaxation structure. In the conventional Hall geometry, the longitudinal and transverse Hall resistivities are a function of the out-of-plane applied magnetic field. (**a**) Schematic of the AHE measurement. (**b**,**c**) Depict the response of the longitudinal and transverse Hall resistivities to the applied field (*H*_*⊥*_) at a temperature of 4 K. (**d**) The longitudinal resistivity as a function of the applied field angle (*θ*) between the applied magnetic field and the $[0\,0\,1]$ axis at a temperature of 4 K with an applied field of 1500 Oe. For the reference (blue), the longitudinal resistance shows a typical hard axis response, in which the resistance increases rapidly at approximately 180°, at which point magnetization reversal occurs. However, for the suspended structure (red), the longitudinal resistance exhibits a smooth behaviour, in which the resistance increase at approximately 180° is not as rapid as in the reference.

Magnetotransport measurements are performed to investigate the anomalous Hall effect. The measurements are performed with an applied magnetic field perpendicular to the free-standing GaMnAs Hall bar structure (H < ± 9 T) using the AC lock-in measurement technique (*I* = 0.1 µA at 17 Hz and 27 Hz). Schematic of the magnetotransport measurement is illustrated in Fig. 2(a). Figure 2(b) plots the longitudinal anisotropic magnetoresistance of both the freely suspended and the reference as the applied magnetic field is swept at *T* = 4 K. Although both the suspended GaMnAs and the reference exhibit similar resistance changes with a high field (*H* > ±1 T), the resistivity drop for a low field shows marked differences: the suspended GaMnAs structure exhibits a smaller flat magnetoresistivity region between jumps than that of the reference. The corresponding transverse Hall responses are plotted in Fig. 2(c). While the anomalous Hall effect response from the reference shows a typical hard axis response, in which the resistivity increases sharply as the magnetization reversal occurs, the response from the suspended structure exhibits a more inclined shape as a reduced hard axis response. Both the AMR and AHE responses suggest that the easy magnetization direction in the suspended structure is more out-of-plane-like, while the reference exhibits typical in-plane-like behaviour, as reported elsewhere^27^. The AMR and AHE responses are further discussed and quantified later, along with the planar Hall measurements.

Further investigation of the strain-relaxation-based magnetic anisotropy changes is conducted by measuring the angle-dependent longitudinal resistivity at *T* = 4 K with an applied field of 1500 Oe, as shown in Fig. 2(d). The longitudinal resistivity in the reference increases rapidly as the angle approaches 180°, where the magnetization reversal occurs, and decreases after the magnetization reversal. In contrast, the suspended structure exhibits a smooth transition in the longitudinal resistivity, which can be attributed to the modification of the magnetic anisotropy from the in-plane direction towards the out-of-plane direction. A similar strain-based magnetic anisotropy modification has been reported in metallic magnetic thin films on a flexible substrate^28^.

**Figure 3:**
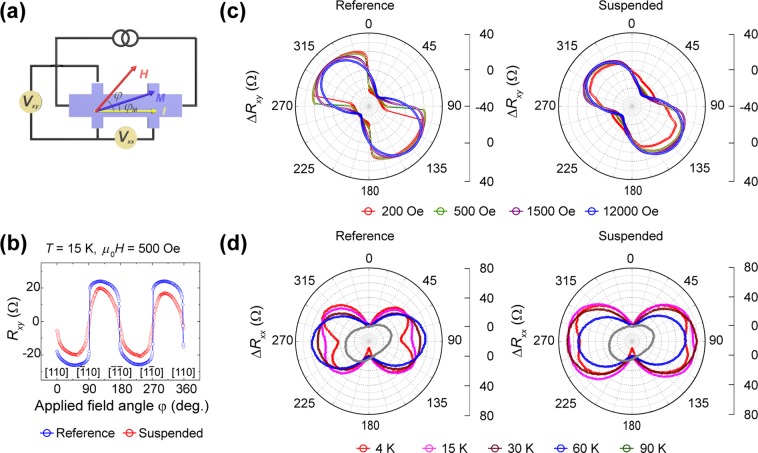
Planar Hall effect measurement in the freely suspended strain-relaxation structure. In the planar Hall geometry, the longitudinal and transverse Hall resistivities are a function of the in-plane applied magnetic field. (**a**) Schematics of the PHE measurement. (**b**) The transverse Hall resistance as a function of the applied field angle (*φ*) between the in-plane applied magnetic field and the injected current direction $[110]$ at a temperature of 15 K with an applied field of 500 Oe. (**c**) Depicts a polar plot of the transverse Hall resistance as a function of *φ* at a temperature of 15 K with applied fields of 200 Oe, 500 Oe, 1500 Oe, and 12000 Oe. Below 500 Oe, the transverse Hall resistance jumping behaviour, attributed to a two-jump sequence of the magnetization: $[100]$ (*φ*_*M*_ ~ ‒45°) → $[010]$ (*φ*_*M*_ ~ 45°) → $[\bar{1}00]$ (*φ*_*M*_ ~ 135°), in the reference appears as a sharp transition, whereas the suspended GaMnAs does not show a sharp transition. (**d**) depicts a polar plot of the longitudinal Hall resistance as a function of *φ* at temperatures of 4 K, 15 K, 30 K, and 90 K with an applied field of 1.2 T. Below 30 K, the resistance change in the suspended sample along $< \bar{1}10 >$ is significantly reduced compared to that in the reference.

The modification of the magnetic anisotropy by strain relaxation is further examined by a planar Hall response (PHR) measurement with an applied magnetic field within the plane of the Hall bar structure, as shown in Fig. 3(a). Figure 3(b) plots the angle-dependent transverse resistance at *T* = 15 K with an applied magnetic field of 500 Oe. While the reference clearly shows switching behaviour attributed to a two-jump sequence of magnetization, [100] (*φ*_*M*_ ~ −45°) → [010] (*φ*_*M*_ ~ 45°) → [100] (*φ*_*M*_~135°), the suspended structure shows a rather semi-sinusoidal shape, and the Hall resistance jumping behaviour disappears. Figure 3(c) depicts the transverse resistance as a function of the applied field angle (*R*_*xy*_ vs. *φ*) at a temperature of 15 K with applied magnetic fields of 200 Oe, 500 Oe, 1500 Oe and 12000 Oe. The resistance jumping behaviour in the reference GaMnAs structure appears as a sharp transition in the plot, whereas the free-standing GaMnAs does not show this sharp transition. Figure 3(d) shows the longitudinal resistance as a function of the applied field angle (*R*_*xx*_ vs. *φ*) at *T* = 4 K, 15 K, 30 K, 60 K, and 90 K with an applied magnetic field of 1.2 T.

In the reference sample, the longitudinal resistance change appears along 90° and 270°, which corresponds to the $< \bar{1}10 >$ direction in GaMnAs, and disappears with increasing temperature. This response exhibits the in-plane cubic anisotropy, which is dominant below ~30 K, as reported by others^29–31^. However, in the free-standing GaMnAs structure, this feature along the $< \bar{1}10 >$ direction in the longitudinal resistance appears only below 15 K. This reduction in the resistance change along $< \bar{1}10 >$ can be attributed to a reduction in the in-plane cubic magnetic anisotropy. To quantify the magnetic anisotropy, the angle dependency of planar Hall resistivity can be analysed by the Stoner-Wohlfarth model^32–34^. At *T* = 10 K and *H* = 1.2 T, fitted values for cubic anisotropy and uniaxial anisotropy are 1151 Oe and 325 Oe for the reference and 380 Oe and 697 Oe, respectively, for the suspended sample. Similarly, from AHE, at *T* = 10 K and *H* = 1.2 T, we find the out-of-plane uniaxial components of the anisotropy fields to be 1767 Oe for the reference and 99 Oe for the suspended sample (see METHODS).

The anomalous Hall effect and planar Hall effect measurements suggest that mechanical strain can be utilized to manipulate the magnetic anisotropy of the GaMnAs epilayer. The origin of this manipulation is the magnetic anisotropy change due to strain. The magnetic easy axis of GaMnAs tends to stay in-plane for compressive strain and out-of-plane for tensile strain^35^. GaMnAs on AlGaAs possesses compressive strain due to the lattice mismatch between the two layers. However, this strain, which is determined during growth, can be modulated by a mechanical strain modulation, resulting in the magnetic anisotropy changes shown in Figs 2 and 3.

The reduction in the in-plane magnetic anisotropy can be attributed to the free-standing Hall bar structure, which is isolated from the GaMnAs layer under compressive strain. Since the Hall bar structure is supported solely by a soft metal electrode as noted above, the isolated GaMnAs structure can be relaxed freely: any possible strain evolved during the restoration of the lattice is absorbed by the soft metal electrode. Without the removal of GaMnAs beneath the metal electrode, the free-standing structure is no longer free from evolving strains: lattice restoration in the beam segment may cause further compression towards both ends of the beam, possibly inducing more compressive strain in semi-rigidly supported Hall probes.

### Magnetotransport measurements in the freely suspended buckled structure

**Figure 4:**
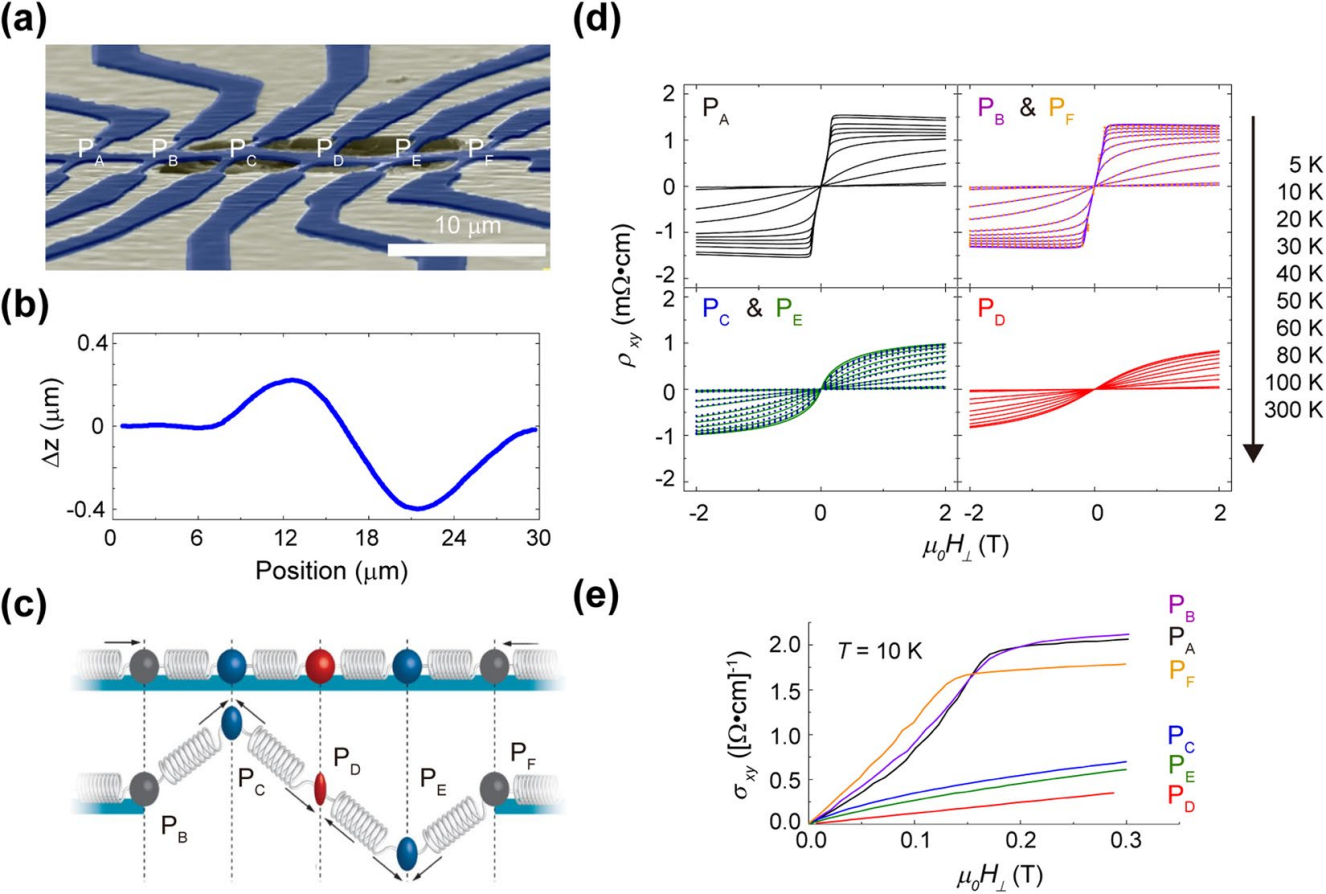
Asymmetrically buckled multiple Hall beam structure. An asymmetrically buckled, micron-sized, GaMnAs Hall beam is realized by micromachining techniques. Along the beam, this structure contains six equally spaced (6 μm) pairs of transverse electrical leads: P_A_, which is the reference probe; P_B_ and P_F_ at the clamped ends of the suspended beam; and P_C_, P_D_, and P_E_ on the buckled GaMnAs beam. (**a**) A false-colour SEM micrograph of the presented sample. (**b**) The height profile of the buckled beam, as measured by a non-contact optical profilometer after processing. Buckling occurs because the underlying AlGaAs epilayer is selectively removed from underneath the GaMnAs/GaAs epilayers, which are compressively strained when grown. (**c**) Depicts our physical model of the buckling process and the resulting strain variations in probes P_C-E_. (**d**) plots the resistivity at various temperatures. The curves represent the reference probe P_A_; the probes P_B_ and P_F_ at the clamped ends; the pairs of probes P_C_ and P_E_ symmetrically placed around the centre of the buckled beam; and finally the central probe P_D_. The reference probe exhibits a prototypical GaMnAs anomalous Hall response. The transverse resistivities at the beam clamps of P_B_ and P_F_ display behaviours similar to the Hall response and similar to each other. The three suspended probes P_C_, P_D_, and P_E_ show significant suppression, which is strongest at the centre of the buckled beam. (**e**) plots *σ*_*xy*_(*H*_*⊥*_), revealing a drastic decrease in the anomalous Hall conductivity in the suspended probes.

Strain induction in the semi-rigidly supported free-standing GaMnAs structure is further investigated in a multiple Hall bar structure. The multiple Hall bar structure is patterned with an identical e-beam lithography technique but is no longer isolated from the GaMnAs layer. The multiple Hall bar, with dimensions (*w* × *l* × *h*) of 2 µm × 30 µm × 175 nm, is designed to contain six equally spaced (*s* = 6 µm) transverse Hall probes (P_A-B,F_, P_C-E_) (with a width of 1 µm), as shown in Fig. 4(a). P_A_ serves as a unsuspended Hall probe (reference), and P_C-E_ serves as suspended Hall probes with semi-rigid supports, while P_B_ and P_F_ are located at the clamped ends. Contrary to the suspended Hall bar structure with a soft metal support, the structure with the semi-rigid support exhibits mechanical buckling phenomena in the second mode, as characterized by non-contact optical microscopy (Fig. 4(b)). At both ends of the suspended part, the “edge structures” are placed where most of the structure is unsuspended (P_B&F_). Hall probes P_C_ and P_E_ are structures with the most vertical displacement, with Hall probe P_C_ buckled up and Hall probe P_E_ buckled down. Hall probe P_D_, placed between P_C_ and P_E_, is an inflection point in the whole structure.

Mechanical buckling is a highly nonlinear phenomenon, sensitive to initial conditions. Buckling of the suspended structure can be explained in terms of the Euler critical load^36^. Using the mechanical buckling model of a beam and the corresponding differential equation, the critical load is ${P}_{cr}={n}^{2}\frac{{{\rm{\pi }}}^{2}EI}{{{L}_{eff}}^{2}},$, where n is a buckling mode and *L*_*eff*_ is the effective length dependent on each end. As we have a beam with both ends fixed, *L*_*eff*_ is half of the total length. The critical stress corresponding to the critical load is *σ*_*cr*_ = *P*_*cr*_*/A* (*A* is the cross-sectional area). With the same dimension as the suspended part of the Hall beam (2 μm × 24 μm × 175 nm for *w* × *l* × *h*), we calculate the critical stress corresponding to the critical load σ_*cr*_ to be 913.9 kPa for the second buckling mode, and the corresponding strain is 0.00107%, which is much smaller than the strain due to the lattice mismatch. Therefore, it is reasonable for the Hall beam structure to buckle.

A simple mass-spring model can be utilized to predict the induced strain in each probe of the buckled structure, with beams as springs and probes as masses (Fig. 4(c)). As noted above, when the GaMnAs beam structure is freely suspended, the beam relaxes and the probes serve as semi-rigid supports. With compression acting on a Hall probe with point symmetry, all the compressions are applied to the Hall probe (P_D_). In contrast, when this point symmetry is broken (P_C&E_), all the compressions cannot be applied, but compressions can be applied that are larger than that before relaxation (P_A_, P_B&F_).

Magnetotransport measurements are performed to test our physical model. For temperatures from 5–300 K, we measure the transverse resistances or the AHE (*ρ*_*xy*_) as a function of the applied magnetic field (*H*_*⊥*_). Figure 4(d) plots the AHE for each probe: the reference probe P_A_, P_B_ and P_F_ at the suspended beam clamps, the off-centre suspended probes P_C_ and P_E_, and the central suspended probe P_D_. P_A_ displays a typical response for GaMnAs, with suppression of the AHE near *T*_*C*_. For the asymmetrically buckled GaMnAs Hall beam, we observe a progressive and symmetric suppression of the anomalous Hall response away from the beam centre. This symmetry in the responses of the off-centre probes can be seen more clearly in Fig. 4(e). Comparing the reference probe P_A_ or the probes at the beam clamps (P_B_ and P_F_) to the off-centre suspended probes (P_C_ and P_E_) and the central suspended probe (P_D_), we see a systematic suppression of *σ*_*xy*_ (*H*_*⊥*_) as we progress towards the probe under the highest strain. The behaviour and values of the strain dependence agree well with theory^20,21,23^.

**Figure 5:**
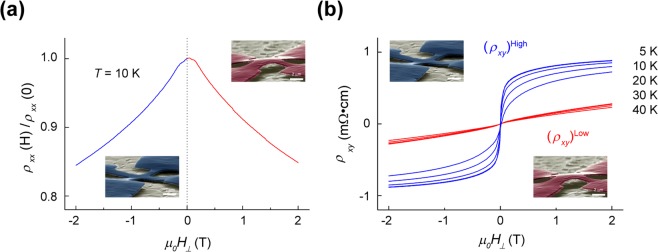
Two-level GaMnAs Hall cross. A simple Hall cross is realized by the same methods used to fabricate the buckled multiple Hall beam structure. For a given structure, we simultaneously measure *ρ*_*xx*_ (*H*_*⊥*_) (**a**) and *ρ*_*xy*_ (*H*_*⊥*_) (**b**). Over repeated thermal cycles, the observed *ρ*_*xx*_ (*H*_*⊥*_) curves are nearly indistinguishable. However, we observe two families of the AHE (*ρ*_*xy*_ (*H*_*⊥*_)) responses. We took SEM micrographs of the Hall cross after cycling to determine whether the beam was buckled or unbuckled (insets in (**b**)). The ‘high’ AHE state (*(ρ*_*xy*_)^*High*^) corresponds to an unbuckled/suspended beam (blue-bordered inset), while the ‘low’ AHE ((*ρ*_*xy*_)^*Low*^) states correspond to a buckled beam (red-bordered inset).

A means to reversibly observe changes in the AHE corresponding to a particular mechanical state would give great credence to our physical model and the strain-dependent and mechanically controlled AHE in GaMnAs. Thus, we realize free-standing simple Hall cross structures with a length of 11 µm and widths of 1 µm and 2 µm. As mentioned previously, mechanical buckling is a nonlinear phenomenon; after processing, we find that some crosses have buckled, while others have not. We then simultaneously measure *ρ*_*xx*_ and *ρ*_*xy*_ as a function of H_⊥_ of the suspended Hall cross at various temperatures below *T*_*C*_, as shown in Fig. 5. For a particular initially buckled sample as verified by SEM, we first see a suppressed AHE response, *(ρ*_*xy*_)^*Low*^. After several thermal cycles between room temperature and *T* = 5 K, the AHE response changed drastically, *(ρ*_*xy*_)^*High*^, while the magnetic field dependence of *ρ*_*xx*_ was unaffected. After the change in the AHE response, we found that the Hall cross mechanical state changed to an unbuckled state, again from SEM (Fig. 5(a,b) insets).

With large differences between *(ρ*_*xy*_)^*High*^ = ‘0’ and *(ρ*_*xy*_)^*Low*^ = ‘1’, highly scalable, robust, non-volatile, low-dissipation binary mechanical sensor elements^17^ can be realized^37^, in which the mechanical state can be robustly read electronically, a non-trivial realization in other proposed nanomechanical memory elements such as those based on carbon nanotubes^17^. Further incorporation of nanomechanical actuators such as electrostatic-driven comb drives allows for dynamic avenues to control axial strain and the buckling process^38^.

## Summary

In summary, we have fabricated three types of suspended GaMnAs Hall bar structures to obtain a varying strain. We observe an in-plane magnetic anisotropy reduction via distinct changes in the PHE and AHE responses as the strain is relaxed in an isolated GaMnAs suspended structure. In the buckled GaMnAs multiple Hall bar structure, transverse transport measurements show a suppression of the AHE that varies symmetrically about the centre of the buckled beam, supporting the interpretation of the strain-induced SOI modifications. We demonstrate a two-level AHE state corresponding to a bi-stable mechanical state of a simple, scalable GaMnAs Hall cross structure.

## Methods

To quantify the magnetic anisotropy field, we assume a coherent rotation of the magnetization in GaMnAs, according to the Stoner-Wohlfarth model with the free energy density described below^39–41^.

1 $$\begin{array}{c}F=\frac{1}{2}M\{-2H[\cos \,\theta \,\cos \,{\theta }_{H}+\,\sin \,\theta \,\sin \,{\theta }_{H}\,\cos (\phi -{\phi }_{H})]\\ \,\,+(4\pi M-{H}_{2\perp }){co}{{s}}^{2}\theta -\frac{1}{2}{H}_{4\perp }{co}{{s}}^{4}\theta \\ \,\,-\frac{1}{2}{H}_{4\parallel }\frac{1}{4}(3-\,\cos \,4\phi ){si}{{n}}^{4}\theta -{H}_{2\parallel }\,{si}{{n}}^{2}\theta {si}{{n}}^{2}\phi \}\end{array}$$

*H*_2⊥_, *H*_4⊥_, *H*_2||_ and *H*_4||_ are the out-of-plane uniaxial and cubic anisotropy fields and the in-plane uniaxial and cubic anisotropy fields, respectively. *θ* is the angle between the magnetization direction and the [001] axis, and *φ* is the azimuthal angle, indicating the direction of the projection of magnetization with respect to the [110] axis.

The in-plane components of anisotropy fields (*H*_2||_, *H*_4||_) can be obtained from the PHR and the free energy F minimum condition (i.e., $\frac{\partial F}{\partial \phi }=0\,{\rm{and}}\,\frac{{\partial }^{2}F}{\partial {\phi }^{2}} > 0$, with $\theta ={\theta }_{H}=\pi /2$) given by

2 $$MH\,\sin (\phi -{\phi }_{H})-\frac{1}{2}M{H}_{2||}\,\sin \,2\phi -\frac{1}{4}M{H}_{4||}\,\sin \,4\phi =0$$

The magnetization angle can be derived from the normalized planar Hall resistance, which is expressed as^41–43^

3 $${R}_{xy}=\frac{k}{t}{M}^{2}\,\sin \,2\phi $$

where *t* is the thickness of the film, *φ* is the angle between the magnetization and current, and k is a constant related to the AMR. By fitting the data measured at 10 K with an applied field of 1.2 T, we calculate the cubic (${H}_{4||}^{ref}$) and uniaxial (${H}_{2||}^{ref}$) anisotropy fields of the reference sample to be 1151 Oe and 380 Oe, respectively, and the cubic (${H}_{4||}^{sus}$) and uniaxial (${H}_{2||}^{sus}$) anisotropy fields of the suspended sample to be 325 Oe and 697 Oe, respectively.

Similarly, we derive the out-of-plane component of the anisotropy fields (i.e., $4{{\rm{\pi }}M}_{eff}=4\pi M-{H}_{2\perp }$.) from the angle dependence of the anomalous Hall resistivity with the free energy F minimum condition (i.e., $\frac{\partial F}{\partial \theta }=0\,{\rm{and}}\,\frac{{\partial }^{2}F}{\partial {\theta }^{2}} > 0$, with $\phi ={\phi }_{H}=\pi /2$) given by:

4 $$\frac{\partial F}{\partial \theta }=\frac{1}{2}M[2H\,\sin (\theta -{\theta }_{H})+4\pi {M}_{eff}\,\sin \,2\theta $$

The magnetization angle can be obtained by normalizing the angle dependence of the anomalous Hall resistance, which is described as^41–43^:

5 $${R}_{xy}=\frac{{\mu }_{0}{R}_{s}}{t}M\,\cos \,\theta $$

where *t* is the thickness of the film, *R*_*s*_ is the anomalous Hall coefficient, and *θ* is the angle between *M* and *I*. By fitting the data measured at 10 K with an applied field of 1.2 T, the out-of-plane uniaxial components of the anisotropy fields $(4{\rm{\pi }}{M}_{eff}=4\pi M-{H}_{2\perp })$ in the reference sample and suspended sample are found to be 1767 Oe and 99 Oe, respectively. This depicts that the out-of-plane uniaxial anisotropy field (*H*_2⊥_) tends to increase as the compressive strain relaxes.

## Data Availability

The datasets generated during and/or analysed during the current study are available from the corresponding author upon reasonable request.
